# Editorial: Gender, civil society and women's movements in the context of Russia's war on Ukraine

**DOI:** 10.3389/fsoc.2025.1735775

**Published:** 2025-12-11

**Authors:** Eva Maria Hinterhuber, Gesine Fuchs

**Affiliations:** 1 Rhine-Waal University of Applied Sciences, Kleve, Germany; 2 Hochschule Luzern, Lucerne, Switzerland

**Keywords:** gender, civil society, women's movement, Ukraine, Russia, war

In February 2022, Russia invaded Ukraine in violation of international law. Since then, there has been war in Europe. Traditionally, feminist analyses are not the focus of public attention in times of war, although war and conflict are deeply gendered. This is true for the different roles ascribed to women and men and non-binary people in times of conflict (and peace): be it in terms of inclusion in the armed forces, be it in the context of forced migration and displacement, or with regard to gender-specific and sexualized violence. For the analysis of Russia's invasion of Ukraine, a gender perspective is also indispensable beyond this (cf. Hinterhuber, 2022): The authoritarian turn in Russia has been accompanied by the establishment of a neo-traditional gender regime. Reference to its values is a crucial part of the justification for Russia's invasion of its neighboring state. Such ideas are also pushed forward in other parts of Europe and worldwide, resulting in repressive gender policies. On the other hand, these tendencies are contrasted by a growing social acceptance of women's and LGBTQIA+ rights. Parts of civil society, gender political movements and organizations throughout the region stand up for these rights: In Ukraine and in the Ukrainian diaspora, the (female-dominated) civil society is at the forefront of providing assistance (cf. Greener, 2024). Russian feminists and LGBTQIA+ activists were among the first targets of repression by the authoritarian system and were from its beginning at the center of protest against external aggression (cf. Edenborg, 2022). In Belarus, activists continue to stand up for the ideals of the uprising in 2020, which was largely carried by women (cf. Shaparaga, 2023). In Poland, the women's movement has long been fighting for reproductive rights and currently also for those of Ukrainian survivors of sexualized war violence (cf. Fuchs and Schweizer, 2025). The broad range of activities of civil society organizations in Germany includes support for queer refugees from Ukraine (cf. e.g. queere-nothilfe-ukraine.de). This cursory look at gender, civil society and gender movements in the context of the Ukrainian war makes clear that long-term political change depends crucially on civil society actors working for democracy and peace—and, as their *conditio sine qua non*, gender justice (cf. True, 2020).

Against this background, the Research Topic “*Gender, civil society and women's movements in the context of Russia's war on Ukraine*” contributes to filling the existing research gaps concerning feminist and gender approaches in studies on political participation, civil society studies as well as peace and conflict studies. It adds to the emerging range of research on Russia's war of aggression against Ukraine through the necessary gender lens. The Research Topic comprises empirical studies about gender relations and civil society in the context of war, contributes to further theory development on gender, peace and war displaying a broad spectrum of gender theoretical approaches, and is of interest for a variety of disciplines beyond political science and sociology as well as transferring knowledge in areas such as social work.

In her contribution, Wood shows how, under Vladimir Putin, masculinist cultural and political practices in Russia have contributed to undermining democracy and civil society and ultimately led to the establishment and consolidation of an autocratic system—as well as paving the way for Russia's invasion of Ukraine. The power structures in contemporary Russia are deeply gendered: Wood highlights the dominance of men in discourse and practice, as well as male power clans that support Putin's rule and the parallel devaluation of the parliament. The simultaneous elevation of female MPs—“Baba Commissars” (Wood)—does not contradict this, as they equally represent a neo-traditional gender regime, the defense of which is used not least to justify the war in Ukraine.

Gradskova, in turn, focuses on Russia's cult of motherhood and analyzes both its gendered individual effects and its significance for Russia's imperialist aspirations. In doing so, she highlights the tension that has always existed in relation to motherhood in various regional contexts: on the one hand, references to motherhood serve to generate symbolic capital, which is regularly used to resist the respective (state) power; on the other hand, authoritarian and totalitarian systems of the present and past regularly refer to motherhood in order to secure their own rule both internally and externally. Contemporary Russia is here no exception: The celebration of mothers and (a heterosexual notion of) family serves as an internal means of promoting the dismantling of gender policy achievements, not least queer rights, and, on the other hand, of securing support for warfare of a militarized state.

From a postcolonial perspective analyzes Kotliuk Russia's full scale-war against Ukraine, taking into consideration its gendered aspects. Kotliuk shows parallels between a Western Orientalist and a Russian imperialist view, which result in the gendered perception of Ukraine as a passive object, a perception with far-reaching consequences. In juxtaposition, the efforts to promote gender equality as well as acceptance and rights of LGBTQIA+ are part of Ukraine's anticolonial struggle. They have even been intensified in recent years, showing the decolonial process in the wake of this war of aggression. Kotliuk's article thus also proves that postcolonial theoretical approaches, which are still underrepresented in research on the region, can yield productive results and contribute to reduce existing gaps.

Russia's full-scale invasion of Ukraine has forced millions of people to flee their homes—a situation to which host countries have responded with different models and approaches. In their article, Ammann Dula and Fuchs focus on the specifics of private, i.e., civil society-based accommodation for refugees, examining this issue using Switzerland as an example. The focus is on power relations, not only between refugees and their hosts, but also between the state and civil society. Based on their empirical research, the authors conclude that care can be provided mutually between refugees and hosts, and that the potentially resulting solidarity can also shift the power imbalance between the state and civil society in favor of the latter.

Russia's war of aggression against Ukraine also has far-reaching implications for research on gender relations in Central Eastern Europe and Eurasia (CEE&E). Like a magnifying glass, power relations among researchers are becoming visible, long-held assumptions are being questioned, and blind spots in the respective perceptions are being exposed. In her article, Johnson addresses very fundamental questions that arise from this. She questions the usefulness of choosing the geopolitical context under discussion here from a gender perspective, problematizes the positionality of researchers, and explores the scope for solidarity among activists from different regions. She concludes that gender research in this field continues to make sense and—by incorporating intersectional, decolonial, and solidarity perspectives, for example—can produce valuable results that go beyond the regional focus.

The editors fully agree with this assessment. Events have gathered pace in the years since February 24, 2022. Russia continues to wage war with extreme brutality, while the international community has often shown itself to be disinterested and divided in its commitment to Ukraine, which is under attack. While certain conflicts remain invisible, other wars have become the focus of international attention and have diverted attention away from Ukraine. At the same time, a rapid rise in populism and authoritarianism threaten the democracies of the Western world. These developments go hand in hand with the weakening of civil society and, as described from various perspectives in the preceding paragraphs, with a massive backlash against gender policy achievements worldwide. This is hardly surprising given that forces such as authoritarianism, nationalism, and populism are connected by sexism, misogyny, and heterosexism (cf. True, 2020).

Over the decades, research on gender, civil society, and gender policy movements in Central Eastern Europe and Eurasia has made valuable contributions in this topical field, touching on sensitive issues, pointing out possible solutions, and repeatedly rebuilding bridges within the complex field of gender studies and the gender policy movement itself.

We hope that the publication of this Research Topic will also contribute to this effort and inspire further discussion and research.
